# 

**Published:** 1888-10-06

**Authors:** 


CONTENTS.

**Physiology and Murder** - - - - - - 1

**Within the Wards** - - - - - - - 2

**The Doctor's Pulpit.**—The Seven Ages of Man: I. The Infant - 3

**In and Out Among the Hospitals.** By A SECRETARY OF TEN YEARS' STANDING - - - - - 4

**Doctors and Chemists.** By DAVID WALSH, L.R.C.P. and S., Edin., etc. - - - - - - 5

**Voyages in Vacation.** II.—By ONE IN SEARCH OF RESTFUL RESTORATIVES - - - - - - 7

**Notes and News** - - - - - - - 8

**Everybody's Page** - - - - - - - 10

**Annotations.**—Sir Morell Ma kenzie's Book.—Other Peop'e's Money.—Examiners Examined at Last - - - - 11

**Military Graveyards and Epitaphs in India.** By AN OLD INDIAN - - - - - - - 12

**False Impressions.** By CAROLINE FOTHERGILL, Author of "An Enthusiast," "A Voice in the Wi derness," etc.—Chapter I.—Mrs. Lennox's Garden Party- - - - 13

**"A Serious Case Against a Hospital."** - - - 14

**Malingering.** By W. CURRAN, A M.D - - - - 15

**National Pension Fund for Nurses** - - - - 16

**Amusements and Relaxation.**—New Puzzle Pr'ze Scheme - r6

**EXTRA SUPPLEMENT.—THE NURSING MIRROR.**

En Passant.—A Doll Show.—Nurses and Uniform.—Massage.—Nurses and Courage.—Di trict Nurses for Surrey - - i

For Queen and Country - - - - - - - ii

The Yellow Fever in Jacksonville - - - - - ii

Lectures - - - - - - - - - ii

Why Indeed! - - - - - - - - iii

Appointments - - - - - - - - iii

Vacancies.—Notes and Queries - - - - - - iii

